# Central command suppresses pressor‐evoked bradycardia at the onset of voluntary standing up in conscious cats

**DOI:** 10.1113/EP090718

**Published:** 2022-11-20

**Authors:** Kei Ishii, Mitsuhiro Idesako, Ryota Asahara, Nan Liang, Kanji Matsukawa

**Affiliations:** ^1^ Human Informatics and Interaction Research Institute National Institute of Advanced Industrial Science and Technology Tsukuba Ibaraki Japan; ^2^ Department of Integrative Physiology Graduate School of Biomedical and Health Sciences Hiroshima University Minami‐ku Hiroshima Japan; ^3^ Cognitive Motor Neuroscience, Human Health Sciences Graduate School of Medicine Kyoto University Sakyo‐ku Kyoto Japan

**Keywords:** baroreflex slope, central command, voluntary standing up

## Abstract

It remains unclear whether cardiac baroreflex function is preserved or suppressed at the onset of standing up. To answer the question and, if cardiac baroreflex is suppressed, to investigate the mechanism responsible for the suppression, we compared the sensitivity of the arterial cardiac baroreflex at the onset of voluntary and passive hindlimb standing in conscious cats. Cardiac baroreflex sensitivity was estimated from the maximal slope of the baroreflex curve between the responses of systolic arterial blood pressure and heart rate to a brief occlusion of the abdominal aorta. The systolic arterial blood pressure response to standing up without aortic occlusion was greater in the voluntary case than in the passive case. Cardiac baroreflex sensitivity was clearly decreased at the onset of voluntary standing up compared with rest (*P* = 0.005) and the onset of passive standing up (*P* = 0.007). The cardiac baroreflex sensitivity at the onset of passive standing up was similar to that at rest (*P* = 0.909). The findings suggest that central command would transmit a modulatory signal to the cardiac baroreflex system during the voluntary initiation of standing up. Furthermore, the present data tempt speculation on a close relationship between central inhibition of the cardiac baroreflex and the centrally induced tachycardiac response to standing up.

## Abstract (highlights)

- **What is the central question of this study?**
  Standing up can cause hypotension and tachycardia. Accumulated evidence poses the simple question, does the cardiac baroreflex operate at the onset of standing up? If the cardiac baroreflex is suppressed, what mechanism is responsible for baroreflex inhibition?
- **What is the main finding and its importance?**
  In cats, we found blunting of cardiac baroreflex sensitivity in the pressor range at the onset of voluntary hindlimb standing, but not of passive hindlimb standing. This finding suggests that central command suppresses pressor‐evoked bradycardia at the onset of standing up, probably in advance, to prevent or buffer orthostatic hypotension.

## 1 INTRODUCTION

In daily life, arterial blood pressure (AP) is reasonably stabilized within a narrow range. The arterial baroreflex, which consists of the aortic and carotid sinus baroreflexes, plays an essential role in the short‐term stabilization of AP (Cowley et al., 1973; Smit et al., 2002). Many physiological studies have clarified in laboratory settings that the arterial baroreflex operates not only at rest but also during steady‐state motor activities (e.g., exercise; Fadel & Raven, 2012; Joyner, 2006). Meanwhile, motor activity in daily life includes dynamic and transient movements, such as standing up, but the arterial baroreflex function during dynamic and transient movements largely remains to be investigated.

It has been reported that standing up causes a transient decrease in AP, reaching a nadir at ∼10 s, and a counter increase in heart rate (HR; Harms et al., 2021; Imholz et al., 1990; Sprangers et al., 1991; Ten Harkel et al., 1990; van Twist et al., 2021). The transient depressor and counter tachycardiac responses to standing up suggest that the arterial cardiac baroreflex continues to operate while standing up (Borst et al., 1982; Doba & Reis, 1974; James & Potter, 1999; Smit et al., 2002). However, those findings were reported based on the ‘averaged’ AP response, and Boddaert et al. (1998) reported that interindividual variability of the systolic AP (SAP) response (e.g., only a pressor response) was notable in the first 30 s after standing up. The reason for this interindividual variability, however, remained unclear.

The interindividual variability of the SAP response to standing up would result from the interindividual way of the cardiovascular regulation. In contrast to the arterial cardiac baroreflex‐dependent regulation during standing, in our laboratory we found that in conscious cats, the arterial cardiac baroreflex was suppressed at the onset of light exercise (static bar‐press exercise and walking), then restored during the exercise (Ishii et al., 2022; Komine et al., 2003). Suppression of the arterial cardiac baroreflex is likely to be driven by central command, a feedforward cardioregulatory signal that descends from higher brain centres in association with motor intention and effort (Matsukawa, 2012; Matsukawa et al., 2013, 2012; Murata et al., 2004). The accumulated evidence encourages us to speculate that, at the onset of standing up, central inhibition of the cardiac baroreflex occurs in the pressor range to blunt the depressor effect by blunting pressor‐evoked bradycardia, while in the depressor range the cardiac baroreflex continues to operate to prompt the restoration of AP by baroreflex tachycardia. Such selective modulation of cardiac baroreflex sensitivity, in addition to feedforward cardiovascular regulation, would be important for the prevention or buffering of possible orthostatic hypotension.

However, the simple question of whether the cardiac baroreflex operates or weakens in the pressor range at the onset of standing up has not yet been answered. Determining whether cardiac baroreflex gain is suppressed and, if so, the mechanism(s) responsible, are important considering that the degree of rapid and/or continuous hypotension in response to standing up is associated with physical performance (Mol et al., 2018), increased risk of falls (Saedon et al., 2016), cardiovascular disease (Ricci et al., 2015; Verwoert et al., 2008) and mortality (Rockwood et al., 2012) and that such poor outcomes might result, in part, from a breakdown of mechanisms regulating the cardiovascular system.

The first objective of this study was to examine whether baroreflex bradycardia is blunted at the onset of voluntary hindlimb standing in conscious cats. To assess cardiac baroreflex function in this transition phase, a brief mechanical occlusion of the abdominal aorta was imposed at the onset of standing up. The second objective was to assess whether central command suppresses cardiac baroreflex function associated with the intention to stand up. The baroreflex function at the onset of voluntary standing up was compared with that at the onset of passive standing up, which was achieved by passively raising the forelimbs of cats to the upper part of a wall by an experimenter. The comparison between voluntary and passive manoeuvres is expected to isolate the factor of ‘motor intention’ and has been used to extract the influence of central command on cardiovascular variables (Ishii et al., 2018, 2012; Matsukawa et al., 2015). The central command‐related component of initial tachycardia to standing up was estimated by examining the relationship between the central inhibition of cardiac baroreflex sensitivity and the centrally induced HR response to standing up. We hypothesized that central command suppresses the cardiac baroreflex function in the pressor range at the onset of voluntary standing up.

## 2 METHODS

### 2.1 Ethical approval

Four female mongrel cats (aged >2 years at the start of the experiment; body weight, 2.7−3.5 kg) were selected because they appeared not to hesitate to rest in a transparent box and to wear a fitted jacket. The cats were reared at another location in Japan (Nagoya Laboratory, Nagoya, Japan) and kept in a temperature‐controlled room (∼25°C) at Hiroshima University. The cats were housed in individual cages and fed once daily. The present study was conducted following the ‘Guiding Principles for the Care and Use of Animals in the Fields of Physiological Sciences’ approved by the Physiological Society of Japan and the Institutional Animal Experimental Committee, Hiroshima University Faculty of Medicine. The experimental protocols were approved by the Committee of Research Facilities for Laboratory Animal Science, Natural Science Centre for Basic Research and Development, Hiroshima University (A13‐75). All experiments complied with the ARRIVE guidelines 2.0 (Percie du Sert et al., 2020) and the principles of animal research (Grundy, 2015) and were performed at Hiroshima University in parallel with other experiments (Ishii et al., 2022).

### 2.2 Training and implantation surgery

The four cats were accustomed to staying in a box on a treadmill and seemed comfortable. Three cats were also trained to become accustomed to running on a motor‐driven treadmill for other experiments. After the habituation and/or training period of treadmill walking, implantation surgery was performed as previously reported (Ishii et al., 2022). Briefly, general anaesthesia was induced by inhalation of 3−4% halothane in an N_2_O (0.5 L/min)–O_2_ (1.0 L/min) gas mixture in a small box and maintained in the range of 1.0−2.5% via an endotracheal tube inserted into the airway. The HR, respiratory movement and rectal temperature were monitored during surgery. The halothane concentration was adjusted if a noxious pinch of the paw or a surgical procedure increased HR and/or respiration or caused withdrawal of the limb. Polyurethane catheters were inserted into the right cephalic vein or left external jugular vein for drug administration and into the right brachial artery for AP measurement. An inflatable cuff occluder (VO‐4; Unique Medical, Japan) filled with sterilized saline was wrapped around the abdominal aorta, which was exposed retroperitoneally. In one cat, muscle activities in the biceps brachialis muscle of the bilateral forelimbs were recorded using a pair of polytetrafluoroethylene (Teflon)‐coated silver wire electrodes. The EMG signal was amplified using a bandpass filter of 50−3,000 Hz. The catheters and lead wires were tunnelled subcutaneously, brought to the exterior of the intrascapular region, then protected by a fitted jacket. After intramuscular injection of 20,000 U/kg benzylpenicillin potassium, the cats were housed in their cages, heated with a heating pad and an external lamp.

The cats were able to stand up, walk and eat food the day after surgery. Antibiotics (100,000 units of benzylpenicillin benzathine; Bicillin Tablets; Banyu Pharmaceutical, Tokyo, Japan) were administered orally for 5−7 days postoperatively. Arterial and venous catheters were flushed and filled with heparinized saline every day. One week postoperatively, HR and AP at rest returned to the presurgical levels, and the cats appeared to be comfortable and ran overground voluntarily.

### 2.3 Experimental protocols

After postoperative days 8−14, cats were repeatedly examined three to four times per week for 1−2 months, in parallel to other experiments (Ishii et al., 2022). This postoperative period appears sufficient for cats to recover enough to move freely (Desrochers et al., 2019; Tsuchimochi et al., 2002).

On the day of the experiment, each cat was placed in a transparent plastic box (length (65 cm) × width (20 cm) × height (35 cm)). The box was kept open for the following tasks. The arterial catheter and EMG wires were connected to a pressure transducer and a light lead cable, respectively. The aortic occluder was connected to a 1.0 ml syringe via an extension tube filled with sterilized saline.

To examine the function of the arterial cardiac baroreflex and the influence of central command on baroreflex function, a brief occlusion (∼3 s) of the abdominal aorta was induced by inflation of the occluder at rest (sitting quietly) and at the onset of voluntary or passive standing up (≤1 s from the onset of movement). Voluntary standing up was defined as voluntary raising of the cat's forelimbs to the upper part of the box induced by showing a meal or a toy or without any stimulus. Passive standing up was performed by wrapping the experimenter's hands around the cat's armpits and passively raising the cat's forelimbs to the upper part of the box. This manoeuvre is expected to reduce or not to require voluntary motor intention and effort (i.e., central command), although it requires muscle contraction of the hindlimbs to a certain extent in order that the cat can stand on its hindlimbs. If voluntary movement for standing up, withdrawal of the limb or unrelated movements occurred during passive standing up, data from the trial were excluded from the analyses. As a control, the cats performed both types of standing up without aortic occlusion. Each task was separated by >3 min of rest and was performed when the cats stood on four limbs. At the end of the experiment, the cats were used in other experiments at Hiroshima University and received a lethal dose of pentobarbital sodium through the cephalic vein.

### 2.4 Data recording

The timing of standing up and aortic occlusion was determined using a manually and electrically marked signal or an EMG signal. The marking signal, AP and EMG were recorded simultaneously on an eight‐channel pen‐writing recorder (Recti‐8K; GE Marquette Medical Systems, Tokyo, Japan) and saved on a computer with an analog‐to‐digital converter (MP100; BIOPAC Systems, Santa Barbara, CA, USA) at a sampling frequency of 1 kHz. Heart rate was estimated from the AP wave. The beat‐to‐beat values of HR, SAP, mean AP (MAP) and diastolic AP (DAP) were recalculated using a software program (AcqKnowledge 3.9.1; BIOPAC Systems).

### 2.5 Data treatment and statistical analysis

Some trials (voluntary standing up, 25 of 102 trials; passive standing up, 3 of 22 trials) were excluded owing to an inadequate pressor response (ΔSAP < 10 mmHg) to aortic occlusion. Baseline HR and AP were defined as the mean values obtained at 10 beats before occlusion at rest. To construct arterial baroreflex curves, HR and AP responses to aortic occlusion were assesed during 20 beats: a span from 10 beats before to 10 beats after the onset of occlusion. The HR response was plotted against every change in SAP of 5 mmHg, taking into account a delay time of two to six beats from the occlusion‐induced pressor response to baroreflex bradycardia, as previously reported (Matsukawa et al., 2016, 2012; Murata et al., 2004). Baroreflex sensitivity for HR was estimated from the maximal slope of the baroreflex curve (Matsukawa et al., 2013, 2012, 2016). The relative baroreflex slope was calculated relative to the mean slope at rest for each cat. SAP‐related values were analysed for the baroreflex slope because the ΔSAP−ΔHR curve seemed to include the saturation point (i.e., lower plateau; Matsukawa et al., 2013), and SAP‐related values are commonly used in daily medical practice to define orthostatic hypotension (van Twist et al., 2021). All variables were averaged over the trials for each cat, then averaged for the four cats. To show intertrial variability, the relative baroreflex slope was also summarized by the number of trials, as previously reported (Ishii et al., 2022; Matsukawa et al., 2013, 2012, 2016).

For control tasks (i.e., standing up without aortic occlusion), pre‐activity values of HR and AP were defined as the mean values for 30 s before the onset of standing up. Cardiovascular changes from pre‐activity values were aligned in each trial based on the timing signal and averaged every 1 s. Given that voluntary standing up can cause various patterns of pressor responses (e.g., a feedforward pressor response and/or standing up‐induced hypotension and/or counter pressor response), the changes in AP and HR were averaged in the range of −5 s before the onset of standing up to the end of standing in each trial. The cardiovascular responses were summarized in trials and in four cats. Similar data treatment was conducted in the passive trials of standing up.

Central command might initiate feedforward regulation of HR with suppression of cardiac baroreflex sensitivity at the onset of standing up. If so, the greater the central command, the greater the centrally induced tachycardiac responses and cardiac baroreflex inhibition. To check whether this idea is possible, we examined the relationship between the degree of central inhibition of cardiac baroreflex sensitivity and the degree of centrally induced tachycardiac response to standing up. The first parameter was defined as the difference in the relative cardiac baroreflex slope between the voluntary and passive cases, and the second parameter was defined as the difference in the HR responses between them. Although the number of data was quite small for correlation analysis, the relationship was analysed using Pearson's correlation coefficient (*r*), not to test the hypothesis of the present study, but to check the possibility of our idea.

For estimation of the sample size needed to test our hypotheses, we used a statistical power analysis tool (G*Power v.3.1.9.6; Kiel University, Germany). Effect sizes were calculated based on previous data from our laboratory (for the number of cats, see Komine et al., 2003; for the number of trials, see Matsukawa et al., 2012). The type 1 error probability and statistical power were set at 0.05 and 0.95, respectively. Assuming these settings, the tool suggested sample sizes of four cats and 14 trials.

The following statistical analyses were chosen based on the results of the normality and equal variance tests. Baseline and pre‐activity values of HR and AP were compared using one‐way repeated‐measures ANOVA with the Holm−Sidak post hoc test. The relative baroreflex slope was compared between conditions (rest, voluntary standing up and passive standing up) using one‐way repeated‐measures ANOVA (for data summarized in cats) or the Kruskal−Wallis one‐way ANOVA on ranks (for data summarized in trials) and post hoc tests (Tukey's or Dunn's method, respectively). In control tasks without occlusion, the average changes in HR and AP were compared between voluntary and passive standing up using an appropriate *t*‐test. The level of significance was set at *P* < 0.05. All statistical analyses were performed with SigmaPlot v.14.0 (Systat Software, San Jose, CA, USA). All variables are expressed as the mean ± SD.

## 3 RESULTS

Table 1 shows that baseline SAP, MAP and HR were lower than the pre‐activity values (SAP, *P* = 0.005 between rest and before voluntary and passive standing up; MAP, *P* = 0.044 between rest and before the voluntary case and *P* = 0.021 between rest and before the passive case; and HR, *P* = 0.034 between rest and before the voluntary case) and that the pre‐activity values were similar between voluntary and passive tasks.

**TABLE 1:** Baseline and pre‐activity values of cardiovascular variables in the four cats

**Variable**	**Rest**	**Before voluntary standing up**	**Before passive standing up**	**ANOVA (*P*‐value)**
SAP (mmHg)	131 ± 15	**139 ± 15** *	**140 ± 14** *	**0.003**
MAP (mmHg)	111 ± 13	**116 ± 12** *	**117 ± 12** *	**0.017**
DAP (mmHg)	91 ± 12	91 ± 11	93 ± 12	0.227
HR (beats/min)	166 ± 13	**178 ± 15** *	173 ± 16	**0.031**

Abbreviations: DAP, diastolic arterial blood pressure; HR, heart rate; MAP, mean arterial blood pressure; SAP, systolic arterial blood pressure.

Values are means ± SD. Significant values (*P* < 0.05) are in bold.

* Significant difference from values at rest (*P* < 0.05).

**FIGURE 1:**
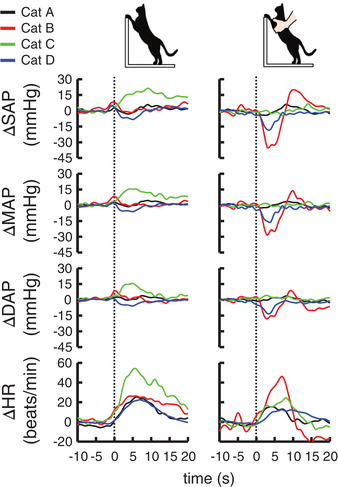
Time courses of arterial blood pressure and heart rate responses to voluntary (left panel) and passive (right panel) standing up without aortic occlusion. The coloured lines indicate the average changes from pre‐activity values in individual cats. Vertical dotted lines indicate the onset of the task. Abbreviations: DAP, diastolic arterial blood pressure; HR, heart rate; MAP, mean arterial blood pressure; SAP, systolic arterial blood pressure

**FIGURE 2:**
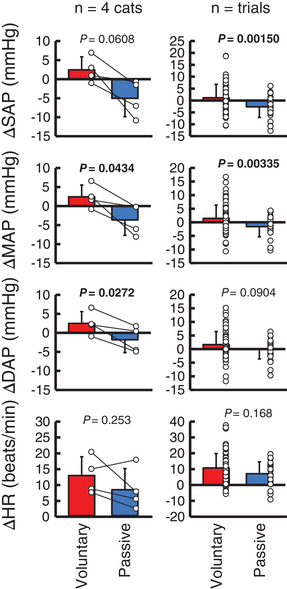
Cardiovascular responses to voluntary and passive standing up without aortic occlusion. Changes in cardiovascular variables from the pre‐activity values were averaged from −5 s before the onset of standing up to the end of standing in each trial. Circles indicate the values for each cat (left panel) or trial (right panel). The number of trials was 79 for voluntary standing up and 29 for passive standing up. Abbreviations are as in the legend to Figure 1. Bar values are the mean ± SD. Significant values (*P* < 0.05) are in bold

Figure 1 summarizes the average responses of AP and HR to standing up without aortic occlusion for each cat. The average duration of standing was 10.1 ± 3.7 s in the voluntary case and 8.7 ± 0.8 s in the passive case. Voluntary standing up increased AP and HR rapidly, whereas this pressor response seemed to be absent at the onset of passive standing up. Figure 2 summarizes the differences in the cardiovascular responses between the voluntary and passive cases. The AP responses averaged among the four cats were greater in voluntary standing up than in passive standing up (ΔSAP, *P* = 0.0608; ΔMAP, *P* = 0.0434; and ΔDAP, *P* = 0.0272), while the HR response was similar between the voluntary and passive cases (*P* = 0.253). Similar differences were observed when summarizing the data in trials (ΔSAP, *P* = 0.00150; ΔMAP, *P* = 0.00335; ΔDAP, *P* = 0.0904; and ΔHR, *P* = 0.168).

**FIGURE 3:**
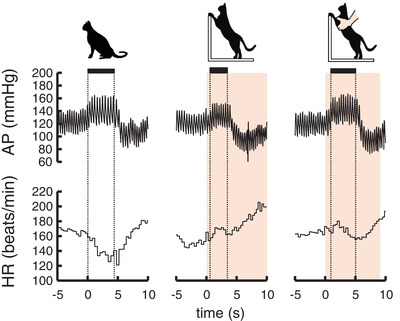
Examples of cardiac baroreflex responses to brief occlusion of the abdominal aorta at rest and at the onset of standing up in a cat. Filed boxes and vertical dotted lines indicate the period during which the occlusion was imposed. Orange areas indicate the period during which standing up was performed. The left, middle and right panels show the baroreflex response at rest and at the onset of voluntary and passive standing up, respectively. Baroreflex bradycardia was blunted only at the onset of voluntary standing up. Abbreviations: AP, arterial blood pressure; HR, heart rate

**FIGURE 4:**
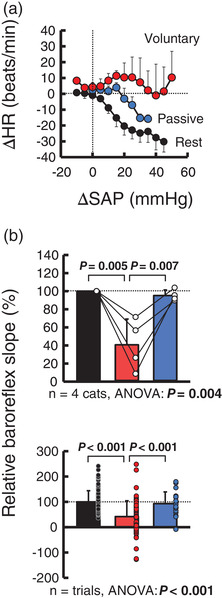
Baroreflex curves between changes in systolic arterial blood pressure (ΔSAP) and change in heart rate (ΔHR) in response to aortic occlusion (a) and maximal slopes of the curves (b) at rest and at the onset of standing up. (a) The baroreflex curves were constructed for every 5 mmHg ΔSAP in four cats. (b) In each cat, the relative baroreflex slope was calculated against the mean value at rest, which was taken as 100%. The upper panel shows the values for four cats; the lower panel shows the values averaged over the number of trials. Blunting of the relative baroreflex slope clearly occurred at the onset of voluntary standing up. The number of trials was 112 for rest (black), 77 for voluntary standing up (red) and 19 for passive standing up (blue). Values are the mean ± SD. Circles indicate the values for each cat (upper panel) or trial (lower panel). Significant values (*P* < 0.05) are in bold

**FIGURE 5:**
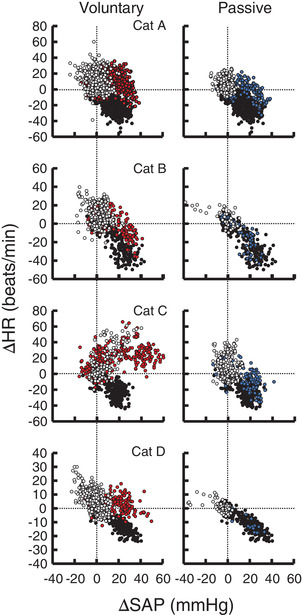
Relationship between systolic arterial blood pressure (SAP) and heart rate (HR) at rest and at the onset of voluntary standing up with and without aortic occlusion in each cat. Individual SAP–HR relationships at rest (black) and at the onset of voluntary and passive standing up with (red and blue circles, respectively) and without occlusion (white circles in both standing up cases) were merged. In cat C, voluntary standing up with and without occlusion caused increases in SAP and HR, indicating suppression of the arterial cardiac baroreflex. In cats A, B and D, the arterial cardiac baroreflex was suppressed in the pressor range but appeared to operate in the depressor range. The cardiac baroreflex slope in the depressor range appears to be similar between voluntary and passive standing up without occlusion

Figure 3 shows the baroreflex bradycardia in response to aortic occlusion at rest and at the onset of standing up in a cat. Baroreflex bradycardia in response to aortic occlusion was clearly suppressed at the onset of voluntary standing up, whereas blunting of cardiac baroreflex sensitivity was absent at the onset of passive standing up. Figures 4 and 5 summarize the averaged and individual baroreflex curves between changes in SAP and HR and the maximal slopes of the curves at rest and the onset of standing up. The relative baroreflex slope was reduced at the onset of voluntary standing up (*P* = 0.005 vs. rest; *P* = 0.007 vs. passive standing up). Passive standing up did not influence the baroreflex slope (*P* = 0.909) compared with that at rest. When the relative baroreflex slope was averaged by the number of trials, a similar reduction of the baroreflex slope was found at the onset of voluntary standing up (*P* < 0.001) compared with those in the other two cases (Figure 4).

**FIGURE 6:**
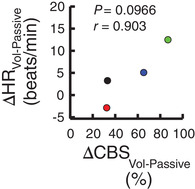
Relationship between the degree of central inhibition of cardiac baroreflex sensitivity and the degree of centrally induced heart rate response in the four cats. The colour of the circles indicates an individual cat, as described in Figure 1. Abbreviations: ΔCBS_Vol‐Passive_, the difference in relative cardiac baroreflex slope between voluntary and passive standing up; HR, heart rate; ΔHR_Vol‐Passive_, the difference in HR response between them. This analysis was not used to test the hypothesis of this study, but to check the possibility of our idea

The central command‐related component of initial tachycardia on standing up was estimated by examining the relationship between the degree of central inhibition of cardiac baroreflex sensitivity (i.e., the difference in relative cardiac baroreflex slope between the voluntary and passive cases) and the degree of centrally induced cardiovascular response to standing up (i.e., the difference in HR responses between the voluntary and passive cases). The degree of central inhibition of cardiac baroreflex sensitivity appeared to be related (*P* = 0.0966, *r* = 0.903) to the degree of centrally induced HR response to standing up (Figure 6).

## 4 DISCUSSION

In this study, we sought to determine whether the cardiac baroreflex operates at the onset of hindlimb standing by using a brief occlusion of the abdominal aorta in conscious cats. Furthermore, we examined the effect of central command on cardiac baroreflex function at the onset of standing up. Our major finding was that cardiac baroreflex sensitivity was blunted at the onset of voluntary standing up, whereas it remained unchanged at the onset of passive standing up. This finding suggests that central command would transmit a signal to switch off the arterial cardiac baroreflex in the pressor range at the onset of voluntary standing up.

It has been reported that the arterial baroreflex plays a crucial role in the counterresponse to hypotension caused by standing up (Borst et al., 1982; Doba & Reis, 1974; Harms et al., 2021; James & Potter, 1999; Smit et al., 2002; van Twist et al., 2021). In contrast, researchers in our laboratory demonstrated suppression of arterial cardiac baroreflex sensitivity during the transition from rest to low‐intensity daily activities, such as bar pressing (Komine et al., 2003) and slow walking (Ishii et al., 2022). To clarify the effect of standing up on cardiac baroreflex sensitivity, a brief aortic occlusion was imposed at the onset of voluntary standing up. We found that cardiac baroreflex sensitivity was blunted in the pressor range at the onset of voluntary standing up (Figure 4). Thus, the pressor‐evoked cardiac baroreflex is likely to be blunted during the transition from rest to low‐intensity voluntary activity, which usually occurs in daily life.

The inhibition of cardiac baroreflex sensitivity at the onset of voluntary standing up would result from interaction between the arterial baroreflex and other neural mechanism(s) activated before and/or during standing up. Central command is the most plausible mechanism responsible for the cardiac baroreflex inhibition in association with voluntary motor intention (Matsukawa et al., 2013, 2012, 2016; Murata et al., 2004). However, it is inevitable that the exercise pressor reflex, which originates from mechano‐ and metabosensitive afferents in skeletal muscles, is activated from the onset of motor activity (Grotle et al., 2020; Kaufman, 2012). To assess the contribution of the exercise pressor reflex to inhibition of the baroreflex, we examined cardiac baroreflex function at the onset of passive standing up. Such a passive manoeuvre is expected to reduce motor intention (i.e., central command) but is accompanied by muscle tension to a certain extent in the limbs moved during the transition phase and muscle contraction in the lower limbs during standing (i.e., exercise pressor reflex). We found that passive standing up did not blunt cardiac baroreflex sensitivity (Figure 4), suggesting that the cardiac baroreflex inhibition is unlikely to result from activation of the exercise pressor reflex. A caveat is needed for data interpretation because the passive manoeuvre cannot develop muscle tension similar to the voluntary case, reducing the exercise pressor reflex, especially at the onset of the manoeuvre. However, our notion is supported by most previous findings that the exercise pressor reflex does not suppress cardiac baroreflex sensitivity (Matsukawa et al., 2012; Murata et al., 2004; Potts & Mitchell, 1998; Smith et al., 2003).

Another plausible explanation for cardiac baroreflex inhibition is recruitment of the cardiopulmonary baroreflex, originating from afferent signals from the atria, ventricles and pulmonary vessels, because standing up and cycling in the upright position increased central venous pressure (Notarius & Magder, 1996; Sprangers et al., 1991). However, Faris et al. (1983) performed careful experiments to avoid several confounding factors and found that in conscious rabbits, the baroreflex sensitivity for HR was not affected by hypervolaemia or by 20% hypovolaemia; the baroreflex sensitivity for systemic vascular resistance was reduced by hypervolaemia and increased by 20% hypovolaemia. Thus, in conscious animals, the naturally stimulated cardiopulmonary baroreflex is likely to alter selectively the vasomotor baroreflex sensitivity but not cardiac baroreflex sensitivity. This notion has been confirmed repeatedly in humans using lower body pressure changes or different postures (Bevegård et al., 1977; Eiken et al., 1994; Fu et al., 2001; Ogoh et al., 2006, 2007). Accordingly, the cardiopulmonary baroreflex would not provide a mechanism for blunting of the cardiac baroreflex sensitivity. The vestibulosympathetic reflex would contribute to cardiovascular regulation during postural change (Doba & Reis, 1974; Ray & Monahan, 2002; Yates et al., 2014). However, this reflex did not explain the voluntary‐specific inhibition of the cardiac baroreflex sensitivity, because the reflex must have been activated during both voluntary and passive standing up.

The above considerations based on the present and previous results suggest that central command suppressed the cardiac baroreflex function in the pressor range at the onset of voluntary standing up. This notion is consistent with accumulated evidence that the arterial baroreflex, especially the aortic baroreflex, is suppressed by central command at the onset of exercise or spontaneous motor activity (Ishii et al., 2022; Komine et al., 2003; Matsukawa et al., 2013, 2012, 2016; Murata et al., 2004). This blunting of cardiac baroreflex sensitivity weakened during a steady state of motor activity (Ishii et al., 2022; Komine et al., 2003). Collectively, the voluntary intention to stand up is likely to trigger central command to suppress pressor‐evoked bradycardia to blunt the depressor effect. Thereafter, it is expected that the central inhibition of the cardiac baroreflex would be switched off during steady‐state standing to stabilize AP, but direct evidence is needed to support this fact accordingly.

One interesting point is whether central inhibition of the cardiac baroreflex results from suppression or shifting of the baroreflex curve. The full range of the cardiac baroreflex curve was unknown in the present study, because no depressor intervention was performed. However, given that standing up without aortic occlusion often imposed depressor responses (Figures 1 and 2), we merged the baroreflex relationships in three conditions: at rest and at the onset of standing up with and without aortic occlusion (Figure 5). The baroreflex relationships in cats A, B and D indicate that voluntary standing up suppressed cardiac baroreflex function in the pressor range, while the cardiac baroreflex appeared to operate in the depressor range. The similar SAP–HR relationship in the depressor range in both voluntary and passive cases supports the functioning of the cardiac baroreflex in the depressor range. This selective inhibition of cardiac baroreflex sensitivity would occur at the level of the nucleus tractus solitarii (Michelini & Bonagamba, 1990) and might result from selective inhibition of the vagal limb while maintaining the sympathetic limb of the cardiac baroreflex. This possibility was confirmed, in part, in decerebrate cats (Matsukawa et al., 2016).

In contrast, the baroreflex relationship in cat C shows a switching off of cardiac baroreflex function at the onset of voluntary standing up, regardless of aortic occlusion, as observed at the onset of spontaneous motor activity in decerebrate cats (Matsukawa et al., 2013, 2012, 2016; Murata et al., 2004). This type of cardiac baroreflex inhibition might result from greater central command, which might strongly inhibit cardiac baroreflex sensitivity and augment centrally induced HR responses, as shown in Figure 6. The above observations urge us to speculate that the manner of central modulation (i.e., suppression or shift) of cardiac baroreflex function might differ depending on the degree of motor intention and effort and/or each individual. The difference in the degree of central inhibition of cardiac baroreflex sensitivity might explain, at least in part, the interindividual variability of SAP responses to standing up.

The question of whether the vasomotor component of the arterial baroreflex is augmented during standing is beyond the scope of this study. However, we believe that the vasomotor baroreflex sensitivity remained unchanged from rest to standing, because the depressor response to stimulation of the aortic nerves or the carotid sinus baroreceptors was similar at rest and during static exercise in conscious cats (Komine et al., 2003) and humans (Fisher et al., 2007). Baroreflex inhibition of renal sympathetic nerve activity occurred to a similar extent both at rest and during spontaneous motor activity in paralysed, decerebrate cats (Matsukawa et al., 2016). Although the previous and present results support our belief, data on renal or lumbar sympathetic nerve activity are lacking in the present study.

This study has several limitations. First, a small number of cats were recruited, because some cats appeared unwilling to stay in the box and wear a fitted jacket. However, the sample size was sufficient for statistical analysis based on sample size calculation, as described in the Methods section. Second, it was technically difficult to control the magnitude of the pressor response caused by a brief occlusion of the abdominal aorta in each of the conditions. However, the evoked pressor response (∼40 mmHg) seemed to cover the range from the operating point to the lower plateau area of baroreflex HR responses (Miki et al., 2003; Norton et al., 1999; Papelier et al., 1994). Third, one might be concerned about the effect of the visual stimulus of the meal or toy on cardiac baroreflex sensitivity; the toy or meal was not shown in the passive trials. The effect of the visual stimulus was estimated by examining the cardiac baroreflex sensitivity during eating, which involved not only chewing activity but also visual stimuli for the meal. We found that eating did not suppress the slope of the cardiac baroreflex (80 ± 8% in four cats, *P* = 0.238, data not shown) in comparison to that at rest, suggesting that neither the visual stimulus of a meal nor the continuous movement (i.e., chewing activity) affected the cardiac baroreflex sensitivity. Fourth, we did not measure several physiological parameters, such as central venous pressure, breathing and muscle activity, that can influence cardiovascular responses to standing up.

In summary, the present study is the first to demonstrate suppression of the arterial cardiac baroreflex in the pressor range at the onset of voluntary but not passive standing up. Considering the present and previous findings (Ishii et al., 2022; Komine et al., 2003), central command would trigger a modulatory signal to the cardiac baroreflex system in association with the intention and effort of daily motor activities.

### 4.1 Perspective and clinical significance

When starting to stand up, central command would cause (selective) inhibition of the cardiac baroreflex and feedforward cardiovascular regulation in advance to prevent or buffer the initial orthostatic hypotension, which is defined as transient hypotension of >40 mmHg SAP and/or >20 mmHg DAP within 15 s of active standing (Freeman et al., 2011; Wieling et al., 2007). Based on large cohort studies, an initial reduction in AP can occur in many old participants (Briggs et al., 2018; Finucane et al., 2014; Romero‐Ortuno et al., 2011; van Twist et al., 2021). Initial orthostatic hypotension might be attributable, in part, to weakened feedforward cardiovascular regulation with ageing and/or inactivity. Romero‐Ortuno et al. (2011) reported that the orthostatic early HR response and SAP recoverability were impaired in frailty. In physically active, healthy older (Imholz et al., 1990) and young (Ten Harkel et al., 1990) participants, brief increases in AP and HR occurred rapidly at the onset of voluntary standing up. Future studies are needed to verify our expectation that exercise training and/or high daily activity would maintain central command function and prevent initial orthostatic hypotension and related adverse outcomes (Mol et al., 2018; Ricci et al., 2015; Rockwood et al., 2012; Saedon et al., 2016; Verwoert et al., 2008).

## AUTHOR CONTRIBUTIONS

Kei Ishii and Kanji Matsukawa conceived and designed the study. All authors acquired the data. Kei Ishii and Mitsuhiro Idesako analysed the data. Kei Ishii, Mitsuhiro Idesako and Kanji Matsukawa interpreted the data. Kei Ishii drafted the manuscript. All authors revised the manuscript, provided intellectual feedback, approved the final version of the manuscript and agree to be accountable for all aspects of the work in ensuring that questions related to the accuracy or integrity of any part of the work are appropriately investigated and resolved. All persons designated as authors qualify for authorship, and all those who qualify for authorship are listed.

## CONFLICT OF INTEREST

None declared.

## Supporting information

Statistical Summary Document

## Data Availability

Data supporting the findings of this study are available from the corresponding author upon reasonable request.
